# ﻿Matching females with males in Chinese species of the strongly dimorphic cockroach genus *Pseudoglomeris* Brunner von Wattenwyl, 1893 (Blattodea, Blaberidae, Perisphaerinae)

**DOI:** 10.3897/zookeys.1255.147028

**Published:** 2025-10-15

**Authors:** Yi-Feng Liu, Tu-Nan Zhou, Sen Chen, Zong-Qing Wang

**Affiliations:** 1 College of Plant Protection, Southwest University, Chongqing 400715, China Southwest University Chongqing China; 2 Key Laboratory of Agricultural Biosafety and Green Production of Upper Yangtze River (Ministry of Education), Southwest University, Chongqing 400715, China Southwest University Chongqing China

**Keywords:** Cockroach, Dictyoptera, field investigation, genitalia, Perisphaerinae

## Abstract

Females and males of six species of *Pseudoglomeris*, P. (P.) sculpta, P. (P.) valida moderata, P. (P.) fallax, P. (P.) beybienkoi, P. (Glomerexis) semisulcata, and P. (G.) angustifolia), are matched by employing both field observation and captive breeding approaches. A key to males of *Pseudoglomeris* from China is provided. The male of P. (P.) sculpta and both sexes of P. (P.) fallax are redescribed and photographs are provided.

## ﻿Introduction

The blaberid cockroach genus *Pseudoglomeris* Brunner von Wattenwyl, 1893 comprises 27 species worldwide (Princis 1964; Beccaloni 2014; Li et al. 2018) and exhibits pronounced sexual dimorphism, as demonstrated in previous studies (Anisyutkin 2003; Li et al. 2018). Females of *Pseudoglomeris* display neoteny, while males exhibit the typical morphology of adult cockroaches. Additionally, some species have been described based on only one sex (Bey-Bienko 1958, 1969; Wang and Che 2011) leading to ambiguous sexual pairing and posing significant challenges in species identification. Li et al. (2018) synonymized five genera: *Glomerexis* Bey-Bienko, 1938, *Trichoblatta* Saussure & Zehntner, 1895, *Kurokia* Shiraki, 1906, *Glomeriblatta* Bey-Bienko, 1950, and “*Corydidarum*” Brunner von Wattenwyl, 1865 with *Pseudoglomeris* Brunner von Wattenwyl and provided a comprehensive summary. Subsequently, Li (2021) redescribed P. (G.) tibetana and resurrected *Glomerexis* from the synonymy of *Pseudoglomeris*, classifying it as a subgenus of *Pseudoglomeris*. To date, 14 species of *Pseudoglomeris* have been recorded from China. However, the sexual pairing of 10 of these 14 species remains unresolved.

Efforts to address the issue of sexual dimorphism in cockroach pairing have been made in previous studies. Roth (1992: 389) attempted to tackle the question of sexual dimorphism pairing in the Australian cockroaches *Laxta* Walker, 1868, stating, “the safest way to be certain that both sexes are correctly associated is to collect them *in copula* (mating)”. With the progress of science and technology, there are now new ways to solve this problem (e.g. DNA barcoding), but morphological methods, field observation, and laboratory rearing are still direct, effective, and convenient.

In this study, we employed field observations and laboratory rearing to resolve sexual dimorphism pairings in *Pseudoglomeris*, successfully pairing six species previously known from only one sex.

## ﻿Materials and methods

### ﻿Morphological study

The specimens examined in this study are deposited at the College of Plant Protection, Southwest University, Chongqing, China (**SWU**). The terminology mainly follows Klass (1998), Roth (2003), and Li et al. (2018). The number of pits on either side of third through seventh tergum is named as the “pit formula”, which serves as a diagnostic character for species identification (Li et al. 2018). A pit formula of 1-1-1-1-2 indicates that third through seventh terga typically has one, one, one, one, and two pits respectively; a pit formula of [3] indicates that each side of the third through seventh tergum has three pits. The abbreviations in this study are as follows: **ce**, cercus; **pp**, paraproct; **st**, stylus; **tc**, typical carinae; **tcl**, typical carina lobe.

The genital segments of the abdomen were dissected and placed in a centrifuge tube containing 10% NaOH, then heated in a water bath for 15 min. After rinsing with distilled water, these segments were observed in glycerin jelly using a Motic K400 stereomicroscope.

Photographs of the specimens were taken using a Leica DFC550 camera which is a part of the Leica M205A stereomicroscope. All photographs were composed using Adobe Photoshop 24.3.0.

### ﻿Male and female pairing

We conducted field observations and laboratory feeding from 5 June 2023 to 28 April 2024. Pairing was first achieved by observing both females and males from the same shelter (under the bark), while nymphs were reared in an artificial environment to facilitate pairing.

## ﻿Results

### ﻿Sexual dimorphism pairings

Six species were successfully paired by biological evidence, listed as follows: Pseudoglomeris (P.) fallax, P. (P.) sculpta, P. (P.) valida moderata, P. (P.) beybienkoi, P. (Glomerexis) semisulcata, and P. (G.) angustifolia.

### ﻿Taxonomy

#### ﻿Genus Pseudoglomeris

Taxon classificationAnimaliaBlattodeaBlaberidae

Brunner von Wattenwyl, 1893

9C9AE7C6-1D31-5D7C-B978-393306C8C999

Pseudoglomeris Brunner von Wattenwyl, 1893: 42, type: Perisphaeria (Perisphaeria) glomeris by original designation—Saussure 1863: 135; Saussure and Zehntner,1895: 37; Li et al. 2018.
“Corydidarum” Brunner von Wattenwyl, 1865 in Beccaloni (2014). Synonymized by Li et al. 2018: 259.
Trichoblatta Saussure & Zehntner, 1895: 44—Kirby 1904: 191; Princis 1964: 207. Type species: Perisphaeria sericea by subsequent designation; synonymized by Li et al. 2018: 259.
Kurokia Shiraki, 1906: 188, type species K. nigra by monotypy—Princis 1964: 207, as synonym of Trichoblatta. Synonymized by Li et al. 2018: 259.
Glomerexis Bey-Bienko, 1938: 123, type species Glomerexis tibetana Bey-Bienko, 1938 by original designation—Wang and Che 2011: 367. Synonymized by Li et al. 2018: 259; Li 2021: 9.
Glomeriblatta Bey-Bienko, 1950: 270, type species Pseudoglomeris planiuscula Brunner von Wattenwyl, 1893: 44, by original designation—Princis 1964: 207, as synonym of Trichoblatta. Synonymized by Li et al. 2018: 259.

##### Diagnosis.

Female neotenic, slightly bulging and oval, integument thick; in male adults, hardening limited to pronotum. Pronotum with typical ventral carinae (Fig. 2D), which end with a lobe or a process, more evident in males. Front femur type C or D. In females and nymphs, abdominal tergites possess one or more pits at sides along tergal furrow (figs 2N, 5–7M, 8L, 9M). Cerci of nymphs and females very short, segments fused into a single segment. Male phallic complex: sclerotizations of cleft phallomere are R1T’, R2’, R3’ and R5’; virga (Fig. 2G) well developed (except subgenus Glomerexis); basolateral sclerite large approximately as wide as hook-like phallomere (Fig. 2G); hook-base sclerite and hook at same side (Li et al. 2018). This genus is similar to *Perisphaerus* Audinet-Serville, 1831, but they can be easily distinguished: the females and nymphs of *Perisphaerus* can roll up into a ball and which body is plump, while in *Pseudoglomeris* they cannot, and the pronotum of males of *Perisphaerus* is more convex than that of males of *Pseudoglomeris*. Two tropical genera of Perisphaerinae—*Frumentiforma* Li, Wang & Wang, 2018 and *Achatiblatta* Li, Wang & Wang, 2018—live in the same habitat as the species of *Pseudoglomeris*. *Achatiblatta* can be distinguished from *Pseudoglomeris* by its small body size; females and nymphs do not bear pits. *Frumentiforma* can be distinguished from *Pseudoglomeris* by the small, slender, cylindrical body and large head, which is wider than ½ of body width.

##### Description.

**Figure 1.**
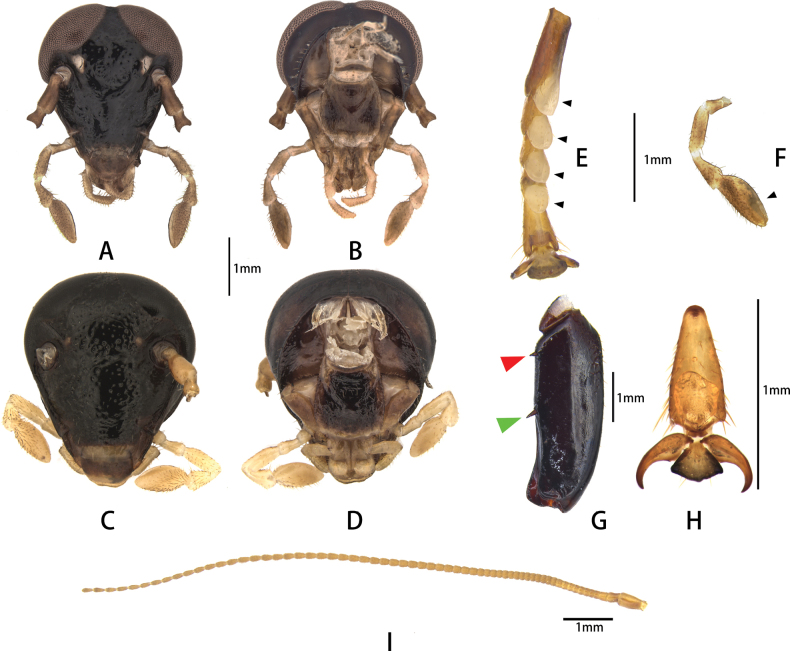
Features of head and leg of Pseudoglomeris (P.) valida moderata. A. Male head, anterior view; B. Male head, posterior view; C. Female head, anterior view; D. Male head, posterior view; E. Tarsi, with arrowheads indicating pulvilli; F. Maxillary palp, with arrowhead indicating apex truncated; G. Femur of hind leg, with red arrowhead indicating basal spine of leg and green arrowhead indicating middle spine; H. Claw; I. Antenna.

**Figure 2.**
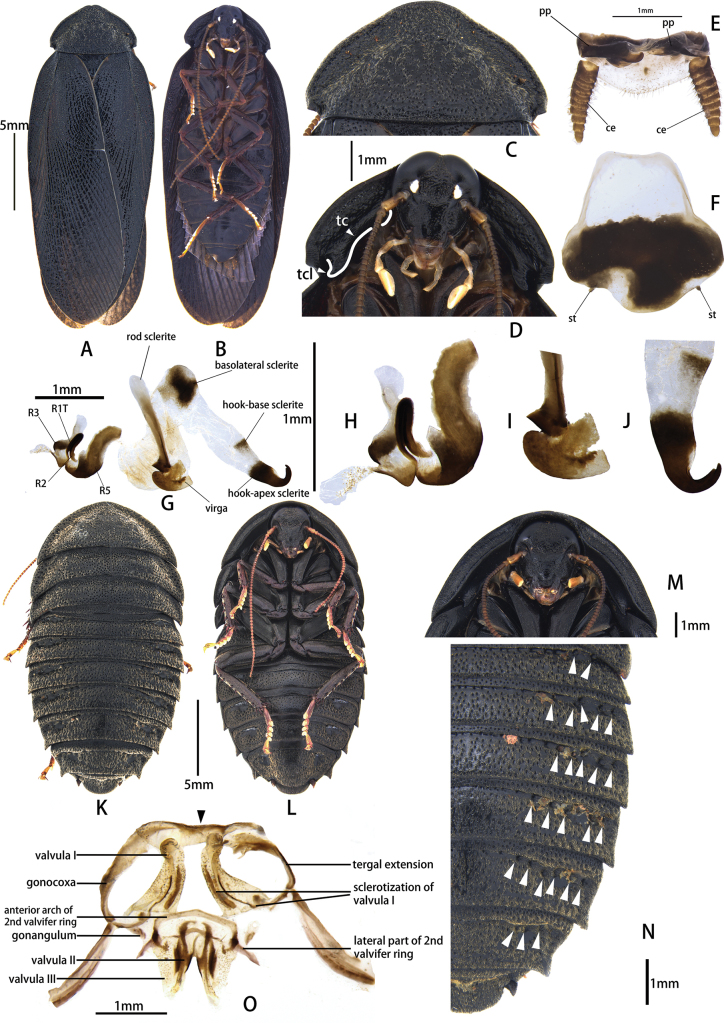
Pseudoglomeris (Pseudoglomeris) sculpta (Bey-Bienko, 1958). A–J. Male: A. Male, dorsal view; B. Male, ventral view; C. Male pronotum, dorsal view; D. Male pronotum, ventral view; E. Supra-anal plate, ventral view; F. Subgenital plate, dorsal view; G. Male genitalia, dorsal view; H. Cleft phallomere, dorsal view; I. Apical half of middle phallomere, dorsal view; J. Hook-like phallomere, dorsal view; K–O. Female: K. Female, dorsal view; L. Female, ventral view; M. Female pronotum, ventral view; N. Female, arrowheads indicate pits; O. Main components of female external genitalia, arrowheads indicate small fold.

**Figure 3.**
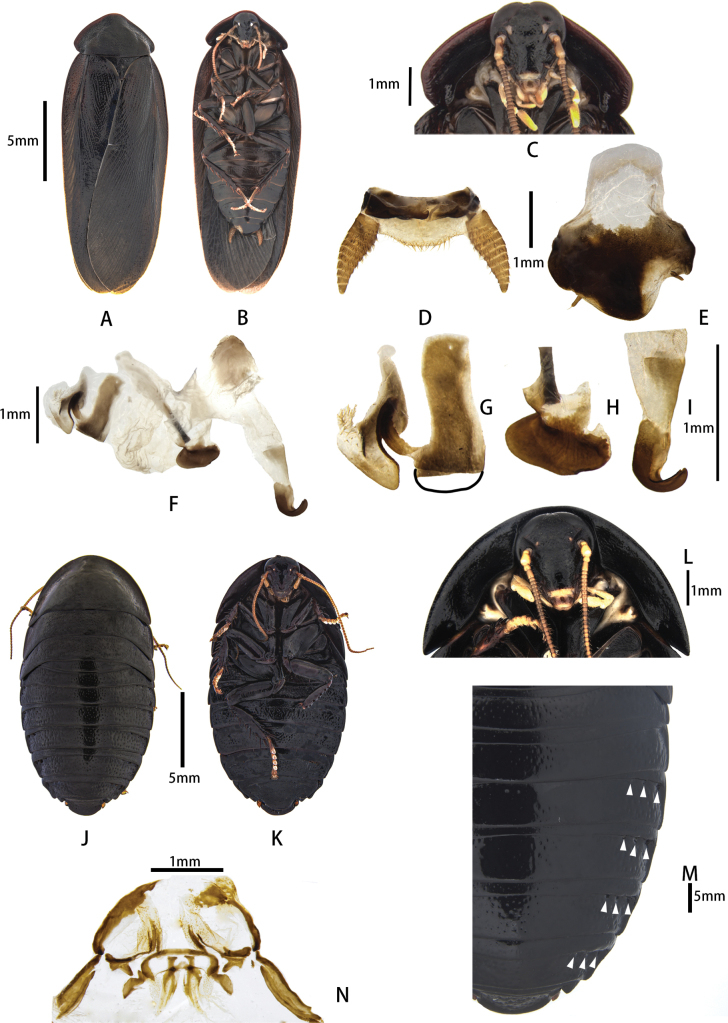
Pseudoglomeris (Pseudoglomeris) valida moderata (Bey-Bienko, 1969) from Guilin. A–I. Male: A. Male, dorsal view; B. Male, ventral view; C. Male pronotum, ventral view; D. Supra-anal plate, ventral view; E. Subgenital plate, dorsal view; F. Male genitalia, dorsal view; G. Cleft phallomere, dorsal view, missing part is outlined with black lines; H. Apical half of middle phallomere, dorsal view; I. Hook-like phallomere, dorsal view; J–N. Female: J. Female, dorsal view; K. Female, ventral view; L. Female pronotum, ventral view; M. Female, arrowheads indicate pits; N. Main components of female external genitalia.

**Male: *head*** (Fig. 1A, B): roundly triangular, as wide as 1/3 width of pronotum, middle of frons exhibits a concavity; surface with slight metallic luster or not; eyes large and reniform, interocular space at vertex narrow; ocelli round or triangular, pale or hardly visible; antennal sockets round, larger than or equal to ocelli; length of antenna ½ length of body, scape and pedicel relatively stout and elongate, flagellar segments short, with terminal flagellar segments more elongated and slender (Fig. 1I); longest maxillary palpomere to the shortest one as 5^th^, 3^rd^, 4^th^, 2^nd^, 1^st^; 5^th^ maxillary palpomere with apex truncated, hollow (Fig. 1F); length of 1^st^ and 2^nd^ segments of labial palpi equal, 3^rd^ labial palpomere elongated and apex truncated. ***Pronotum***: transverse, anterior margin curved, posterior margin straight, disc convex; dorsal surface with obvious punctations or not; ventrum with typical carinae at sides, which end with a lobe or a process. ***Tegmina***: fully developed, reduced, or absent; in macropterous form, elongated oval, venation with lots of obvious cells composed by longitudinal veins and numerous cross-veins, color the same as dorsal surface of pronotum; in brachypterous form, tegmina ovate, leathery, covering ½ of abdomen, with lots of obvious cells, color the same as dorsal surface of pronotum. ***Legs***: dark red to black, sometimes slightly metallic; front femur type C1 or sometimes C0 or D; hind and middle legs with or without one spine on posterior margin of femur, with or without a basal spine (Fig. 1G); tarsomeres 1–4 with pulvilli (Fig. 1E), tarsal claws symmetrical, arolia present (Fig. 1H). ***Abdomen***: soft, color similar to pronotum; 8^th^ abdominal sternite largely covered by 7^th^ sternite, only posterior margin exposed; subgenital plate with a large membranous area on right side (Fig. 3K); styli present or absent on both sides; paraprocts obvious and apex rounded, left one stouter than right one. ***Genitalia*** (Fig. 2G–J): male phallic complex consisting of three major parts, namely, cleft phallomere, middle phallomere, and hook-like phallomere; sclerotizations of cleft phallomere are R1T, R2, R3 and R5; R1T and R2 forming hairpin sclerite, R5 and R3 oval, upper ½ of R5 and R3 gradually narrowing; middle phallomere composed of rod sclerite and virga or only rod sclerite; rod sclerite virgulate, virga oval, linking rod sclerite by thin sheet; hook-like phallomere containing three sclerites: basolateral sclerite flaky; hook-base sclerite triangular or stripe-shaped; terminal part of hook-apex sclerite bifurcate or not.

**Female: *head***: rounder than male, surface with slight metallic luster or not; width of head 1/3 width of pronotum; middle of frons exhibits a concavity or not; eyes large and reniform, interocular space on vertex wider than males; ocelli round or triangular, smaller than that of males, pale or hardly visible; antennal sockets round, bigger than ocelli; length and shape of antenna, labial palpus and maxillary palpus similar to males (Fig. 1A–D). ***Pronotum***: transverse, anterior margin curved, posterior margin truncated, dorsal surface with obvious punctations or not; thicker than in males; disk convex; ventral pronotum with typical carinae at sides (Fig. 2M). Tegmina and hindwing absent; abdomen integument hardened, sometimes slightly metallic; pits usually present on both sides of third through seventh tergum. ***Abdomen***: 8^th^ and 9^th^ abdominal tergites concealed, only posterior margins exposed. Cerci of females very short, with only one segment. Supra-anal plate transversely oval, anterior margin truncated, posterior margin curved; dorsal surface with punctations. ***Genitalia*** (Fig. 2O): Two tergal extensionsstraight, linking supra-anal plate and major part of genitalia; two gonocoxae banded and curved, extremity of gonocoxae approaching each other; gonangulum rectangular or trapezoidal, one or two protrusions present on apical edge; valvula I bended, extremity of valvula I rounded, containing two sclerites internally (sclerotization of valvula I); valvula II bended, valvula III triangular; basal part of valvula II and valvula III enlarged; anterior arch of 2^nd^ valvifer ring shallowly arched; lateral part of posterior lobe of 2^nd^ valvifer ring short, tip enlarged and arrowhead-shaped.

##### Remarks.

The term “Corydidarum” was initially regarded as a generic name in the cockroach species file (Beccaloni 2014) and later classified as a synonym of *Pseudoglomeris* (Li et al. 2018). Poggi (2023) clarified that “Corydidarum” is not a scientific name. Brunner von Wattenwyl (1865) wrote “Corydidarum gen. et sp. nov.”, indicating that Perisphaeria (Blepharodera) sericea Saussure, 1863 should be regarded as a new species within a yet-to-be-described genus of the family Corydiidae (Poggi 2023). The term “Corydidarum”, meaning “of the Corydidae [Corydiidae]”, is merely the Latin plural genitive (correctly ending in -*arum*) of the family name Corydiidae.

### ﻿Key to the males of Chinese *Pseudoglomeris* species except for *P. nigra* (Shiraki, 1906)

(* indicates unconfirmed sexual paring)

**Table:** 

1	Apterous or brachypterous; virga sclerite absent	**2 (P. (Glomerexis))**
–	Wings fully developed, virga sclerite present	**4**
2	Apterous	P. (Glomerexis) tibetana (Bey-Bienko, 1938)*
–	Brachypterous	**3**
3	Subgenital plate with a deep narrow incision near right stylus (Fig. 6E)	**P. (Glomerexis) semisulcata Hanitsch, 1924**
–	Right part of subgenital plate intact without a deep narrow incision near right stylus	**P. (Glomerexis) angustifolia (Wang & Che, 2011)**
4	Surface of body with dense hair (Fig. 2C)	**5**
–	Surface of body without dense hair	**6**
5	Posterior lateral angles of pronotum with notches (Fig. 2A, K)	**P. (P.) sculpta (Bey-Bienko, 1958)**
–	Posterior lateral angles of pronotum smooth	**P. (P.) aerea (Bey-Bienko, 1958)**
6	Pronotum with very dense punctations, each punctuation with an obvious short hair	**P. (P.) planiuscula Brunner von Wattenwyl, 1893** *
–	Pronotum with punctations, not each punctuation with an obvious short hair	**7**
7	Pronotum broad and oval, humeral angle situated at middle of lateral margin of the pronotum	**P. (P.) magnifica Shelford, 1907**
–	Pronotum comparatively narrow, humeral angle situated at posterior 1/3 of lateral margin of pronotum	**8**
8	Pronotum and head with bronze luster	**9**
–	Pronotum and head brown or black	**10**
9	Pronotum and head exhibit strong bronze-like gloss (Fig. 5A–C)	**P. (P.) beybienkoi (Anisyutkin, 2003)**
–	Bronze-like gloss weak on the pronotum, strong on the head	P. (P.) montshadskii (Bey-Bienko, 1969) and P. (P.) dubia Hanitsch, 1924* **(indistinguishable based on male morphology)**
10	Pronotum and abdomen reddish-brown, leg and tegmina yellow; color of area between vertex and anterior edge of median concavity on frons lighter than other parts of frons and gena	***P. (P.) montana* Li, Wang & Wang, 2018**
–	Pronotum, abdomen, leg and tegmina black or dark brown, color of head uniform	**11**
11	R5 wide (Fig. 3G)	***P. (P.) valida moderata* (Bey-Bienko, 1969)**
–	R5 slender (Fig. 4G)	***P. (P.) fallax* (Bey-Bienko, 1969)**

### ﻿Key to the Females of Chinese *Pseudoglomeris* species except for *P. nigra* (Shiraki, 1906)

(* indicates unconfirmed sexual paring)

**Table:** 

1	Surface of body with dense hair (Fig. 2K)	**2**
–	Surface of body without dense hair	**3**
2	Dorsal surface of body rough, with distinct punctations and prominent engravings; pit formula [5, 6, or 7]] (Fig. 2N)	***P. (P.) sculpta* (Bey-Bienko, 1958)**
–	Dorsal surface of body smooth, without prominent engravings, punctations not distinct; pit formula [2–3]	***P. (P.) aerea* (Bey-Bienko, 1958)**
3	Pit formula [4–6]	***P. (P.) magnifica* Shelford, 1907**
–	Pit formula [3] or less	**4**
4	Lateral sides of mesonotum and metanotum bulging	**5**
–	Lateral sides of mesonotum and metanotum flat	**6**
5	Central part of dorsal surface of thorax reddish	***P. (P.) montshadskii* (Bey-Bienko, 1969)**
–	Color of dorsal surface of thorax uniform	P. (P.) dubia Hanitsch, 1924*
6	Pit formula [3]	**7**
–	Pit formula [1–2]	**9**
7	Pronotum with very dense punctations, each punctuation with an obvious short hair	P. (P.) planiuscula Brunner von Wattenwyl, 1893*
–	Pronotum with punctations, punctuation with or without inconspicuous short hair	**8**
8	Tarsi brown, tibia of both the middle and hind legs with a sharp projection at the apex of the ventral surface (Fig. 7K)	**P. (G.) tibetana (Bey-Bienko, 1938)** *
–	Tarsi orange-yellow, middle and hind legs without a sharp projection at the apex of the ventral surface	***P. (P.) valida moderata* (Bey-Bienko, 1969)**
9	Surface of body bronze	**10**
–	Surface of body black or burgundy (Figs 6I, J, 7I, J)	**P. (G.) semisulcata Hanitsch, 1924 and P. (G.) angustifolia (Wang & Che, 2011) (indistinguishable based on female morphology)**
10	Pronotum smooth; pit formula [2]	***P. (P.) montana* Li, Wang & Wang, 2018**
–	Pronotum with dense punctations (Fig. 5J); pit formula [1] (Fig. 5M)	***P. (P.) beybienkoi* (Anisyutkin, 2003)**

### ﻿Subgenus Pseudoglomeris (Pseudoglomeris) Brunner von Wattenwyl, 1893

#### ﻿ Pseudoglomeris (Pseudoglomeris) sculpta

Taxon classificationAnimaliaBlattodeaBlaberidae

(Bey-Bienko, 1958)

2B0151EF-BAC5-5B18-82E4-DF3D954D6CB1

Fig. 2

Glomeriblatta sculpta Bey-Bienko, 1958a: 683 (in Russian); 1958b: 594 (English version).
Trichoblatta sculpta —Anisyutkin, 2003: 71.
Pseudoglomeris sculpta (Bey-Bienko, 1958)—Li et al. 2018: 266.
Pseudoglomeris (Pseudoglomeris) planiuscula (Brunner, 1893)—Li 2021: 9.

##### Material examined.

China • 1 male & 1 female, Yunnan Province, Ning’er Hani and Yi Autonomous County, Pu’er Mountain; 1638 m elev.; 23.0675°N, 101.0299°E; 22 Mar. 2024; Yi-Feng Liu, Peng-Hui Hu, Sen Chen, Tu-Nan Zhou leg; SWU-B-BB103-1 to 2.

##### Supplementary description.

**Male.** The body is indigo-black, with sparse, fine, yellow hairs on the pronotum (Fig. 2C). The surface of the legs is sepia-colored. The posterior lateral angles of the pronotum have a notch. The hind and middle legs lack spines along the posterior margin of the femur and the base.

##### Diagnosis.

The male of this species is similar to P. (P.) aerea (Bey-Bienko, 1958), which also has fine yellow hair on the pronotum. However, it can be distinguished by its indigo-black body surface, the notched posterior lateral angles of the pronotum, and weakly bifurcate terminal part of the hook-apex sclerite. The females of this species can be easily distinguished from P. (P.) aerea by the rough dorsal surface and their pit formula, [5, 6 or 7] in P. (P.) sculpta and [1 or 2] in P. (P.) aerea.

#### ﻿ Pseudoglomeris (Pseudoglomeris) validamoderata

Taxon classificationAnimaliaBlattodeaBlaberidae

(Bey-Bienko, 1969)

5C2E6A6D-22AC-5C88-80AB-C4866401E3FF

Fig. 3

Trichoblatta valida moderata Bey-Bienko, 1969: 836 (in Russian); 1970: 530 (English version)—Anisyutkin 2003: 71.
Pseudoglomeris valida moderata (Bey-Bienko, 1969)—Li et al. 2018: 269. Pseudoglomeris (Pseudoglomeris) valida moderata (Bey-Bienko, 1969)—Li 2021: 9.

##### Material examined.

China • 1 male & 2 females; Guangxi Zhuang Autonomous Region, Guilin; 21 Apr. 2024; Hao-Fei Fan leg; SWU-B-BB57-3 to 5 • 3 males; Guizhou Province, Duyun; 9 Nov. 2023; Hao Cui leg; SWU-B-BB60-1 to 3 • 1 female; Yunnan Province, Honghe Hani and Yi Autonomous Prefecture, Pingbian, Dawei Mountain; 2020; Lu Qiu leg; SWU-B-BB54-1.

##### Supplementary Description.

**Male.** Body brown-black, antennae and tarsi brownish-yellow. R5 strong and wide; hook-apex sclerite strong.

##### Diagnosis.

The male of this species is similar to P. (P.) fallax (Bey-Bienko, 1969). However, P. (P.) valida moderata can be distinguished by its wider R5. The females can be differentiated by their pit formula, namely, [1] of P. (P.) fallax and [3] of P. (P.) valida moderata.

##### Remarks.

The drawings of this species by Li et al. (2018: fig. 26) were not published with the intended grayscale; therefore, the sclerotization of the female external genitalia appears paler than the original version. This is a problem also with Li et al.’s (2018) figure 40.

#### ﻿ Pseudoglomeris (Pseudoglomeris) fallax

Taxon classificationAnimaliaBlattodeaBlaberidae

(Bey-Bienko, 1969)

AB78D08C-6086-528E-B601-1A338F39B304

Fig. 4

Trichoblatta fallax Bey-Bienko, 1969: 836 (in Russian); 1970: 531 (English version)—Anisyutkin 2003: 69.
Pseudoglomeris fallax (Bey-Bienko, 1969)—Li et al. 2018: 270.
Pseudoglomeris (Pseudoglomeris) fallax (Bey-Bienko, 1969)—Li 2021: 9.

##### Material examined.

China • 1 male; Fujian Province, Wuyi Mountain; 27.3943°N, 118.0134°E, 7 Jul. 2013; Shi Yan leg; SWU-B-BB62-1 • 1 female; Fujian Province, Fuding, Taimu Mountain; 27.0637°N, 120.1035°E; 19 Jul. 2013; Shi Yan leg; SWU-B-BB62-2 • 1 male & 1 female; near Hubei Province Shengnongjia Wangyue Hotel; Jul. 2023; Wei Han leg; SWU-B-BB61-1 to 2 • 1 female; Sichuan Province, Huaying, Gaodeng Mountain; 1 Jan. 2024; Yi-Feng Liu leg; SWU-B-BB65-1.

##### Supplementary description.

**Figure 4.**
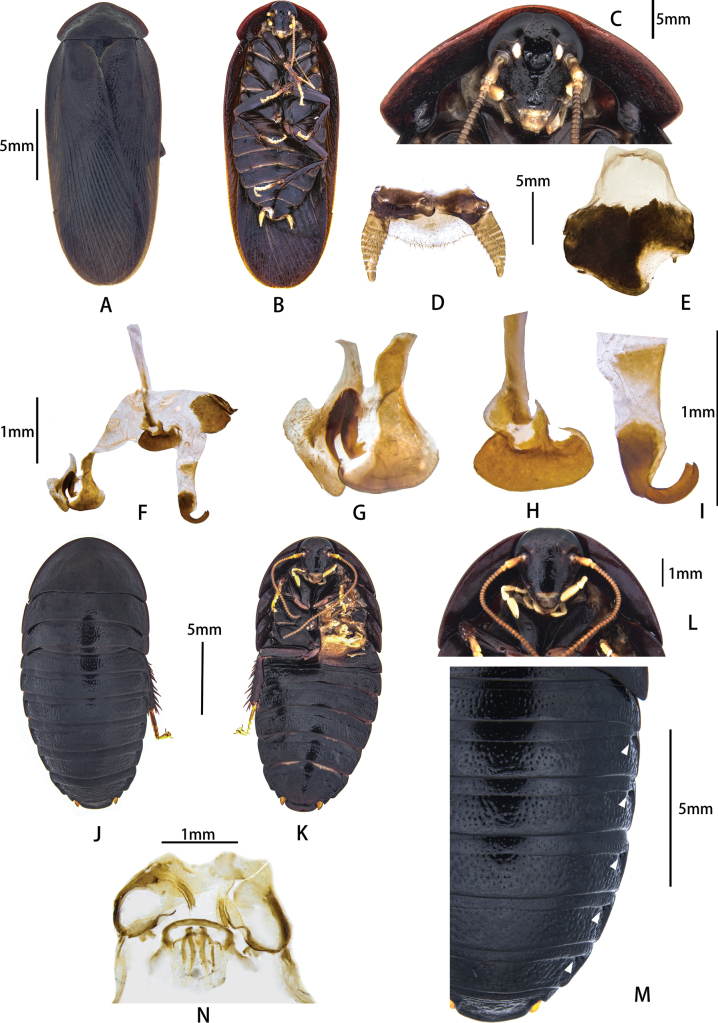
Pseudoglomeris (Pseudoglomeris) fallax (Bey-Bienko, 1969). A–I. Male from Wuyi Mountain: A. Male, dorsal view; B. Male, ventral view; C. Male pronotum, ventral view; D. Supra-anal plate, ventral view; E. Subgenital plate, dorsal view; F. Male genitalia, dorsal view; G. Cleft phallomere, dorsal view; H. Apical half of middle phallomere, dorsal view; I. Hook-like phallomere, dorsal view; J–N. Female from Taimu Mountain: J. Female, dorsal view; K. Female, ventral view; L. Female pronotum, ventral view; M. Female, arrowheads indicate pits; N. Main components of female external genitalia.

**Male. *Coloration***: head and face black; ocelli pale; antennal sockets white; antenna brown; labial palp and maxillary palp orange-yellow. Pronotum and tegmina black or dark brown. Legs black; tarsi orange-yellow; pulvilli and arolia white. Abdomen black or dark brown. ***Head***: roundly triangular, as wide as 1/3 width of pronotum, middle of frons exhibits a concavity; surface with slight metallic luster or not; eyes large and reniform, interocular space at vertex narrow; ocelli round or triangular; antennal sockets round, larger than or equal to ocelli (Fig. 4C). ***Pronotum***: transverse, anterior margin curved, posterior margin flattened, disc convex; dorsal surface with punctations; ventrum with typical carinae at sides, which end with a lobe (Fig. 4A, C). ***Tegmina***: fully developed, elongate oval, extending beyond end of abdomen; venation with lots of obvious cells composed by longitudinal veins and numerous cross-veins (Fig. 4A). ***Legs***: front femur type C1; hind and middle legs each have one spine on posterior margin of femur and base. ***Abdomen***: soft; subgenital plate with a large membranous area on right side; styli present on both sides (Fig. 4E); paraprocts obvious and apex rounded, left one stouter than right one (Fig. 4D). ***Genitalia***: distal half of R5 slightly slender; terminal part of hook-apex sclerite bifurcate (Fig. 4F–I).

**Female. *Coloration***: head and face black; ocelli pale or invisible; antennal sockets white; antenna brown; labial palp and maxillary palp orange-yellow. Pronotum and tegmina black or dark brown. Legs black; tarsi orange-yellow; pulvilli and arolia white. Abdomen black or dark brown. ***Head***: rounder than male; width of head 1/3 width of pronotum; middle of frons exhibits a concavity but not obvious; eyes large and reniform, interocular space on vertex wider than in males; ocelli round or triangular, smaller than that of males; antennal sockets round, bigger than ocelli; length and shape of antenna. ***Pronotum***: transverse, anterior margin curved, posterior margin truncate, dorsal surface with punctations but almost smooth; thicker than in males; disk convex; ventral pronotum with typical carinae at sides (Fig. 4J, L). Tegmina and hindwing absent. ***Legs***: front femur type C1; hind and middle legs each have one spine on posterior margin of the femur and base. ***Abdomen***: integument hardened (Fig. 4J, K); pit formula [3] (Fig. 4M). Cerci very short, with only one segment. Supra-anal plate transversely oval, anterior margin truncate, posterior margin curved; dorsal surface with punctations (Fig. 4M).

##### Diagnosis.

Refer to the diagnosis of P. (P.) valida moderata.

#### ﻿ Pseudoglomeris (Pseudoglomeris) beybienkoi

Taxon classificationAnimaliaBlattodeaBlaberidae

(Anisyutkin, 2003)

FB32B0DC-34B5-5066-85B4-2AFD063511D9

Fig. 5

Trichoblatta beybienkoi Anisyutkin, 2003: 65.
Pseudoglomeris angustifolia (Wang & Che, 2011)—Li et al. 2018: 272. Pseudoglomeris (Pseudoglomeris) beybienkoi (Anisyutkin, 2003)—Li 2021: 9.

##### Material examined.

China • 3 males & 6 females; Yunnan Province, Dali Bai Autonomous Prefecture, Weishan Yizu Huizu Autonomous County, Weibao Mountain; 2286 m elev.; 25.1801°N, 100.3499°E; 22 Apr. 2024; Yi-Feng Liu, Peng-Hui Hu, Sen-Chen, Tu-Nan Zhou leg; SWU-B-BB52-2 to 10 • 5 males & 1 female; Guizhou Province, Guiyang, Guiyang Forest Park; 26.55°N, 106.76°E; 5 Mar. 2019; Qiu Lu, Wen-Bo Deng, Sen Chen, Shu-Ran Liao, He-Jia Jun, Yi-Shu Wang, Jin-Du Ting & Li-Bo Yan leg; SWU-B-BB53-1 to 6 • 1 male & 4 females; Yunnan Province, Honghe Hani and Yi Autonomous Prefecture, Gejiu, Yin Mountain; 2063 m elev.; 23.3817°N, 103.1690°E; 7 Feb. 2024; Yi-Feng Liu leg; SWU-B-BB53-1 to 5 • 1 male; Yunnan Province, Xinping Yi and Dai Autonomous County, Jinshan Primeval Forest, 10 May 2016; Lu Qiu, Zhi-Wei Qiu leg; SWU-B-BB52-1.

**Figure 5.**
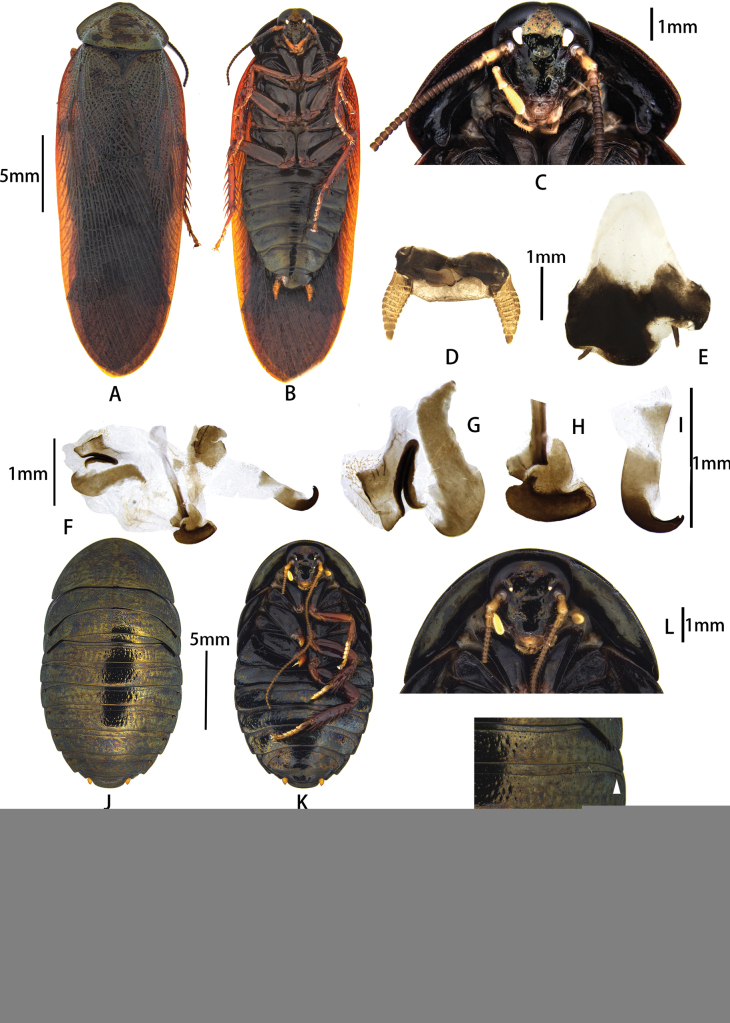
Pseudoglomeris (Pseudoglomeris) beybienkoi (Anisyutkin, 2003) from Guiyang. A–I. Male: A. Male, dorsal view; B. Male, ventral view; C. Male pronotum, ventral view; D. Supra-anal plate, ventral view; E. Subgenital plate, dorsal view; F. Male genitalia, dorsal view; G. Cleft phallomere, dorsal view; H. Apical half of middle phallomere, dorsal view; I. Hook-like phallomere, dorsal view; J–M. Female: J. Female, dorsal view; K. Female, ventral view; L. Female pronotum, ventral view; M. Female, arrowheads indicate pits; N. Main components of female external genitalia.

##### Supplementary description.

**Male.** The apex of R5 extends upwards and to the right.

##### Diagnosis.

The females of this species are similar to P. (P.) montana and P. (P.) magnifica, but they can be differentiated by their pit formula, [1] in P. (P.) beybienkoi, [2] in P. (P.) montana and [4–6] in P. (P.) magnifica. The male of this species resembles P. (P.) magnifica, but they can be distinguished by the broad and oval pronotum of P. (P.) magnifica.

##### Remarks.

The drawings of this species by Li et al. (2018: fig. 30) were not published with the intended grayscale; therefore, parts of the sclerotizations appear oddly darker than the original version.

### ﻿Subgenus Pseudoglomeris (Glomerexis) Bey-Bienko, 1938

#### ﻿ Pseudoglomeris (Glomerexis) semisulcata

Taxon classificationAnimaliaBlattodeaBlaberidae

Hanitsch, 1924

DEFBB6D4-1F4B-5DC9-8571-8F1D1DF4DE5E

Fig. 6

Pseudoglomeris semisulcata Hanitsch, 1924: 338, locality China, Yunnan.
Trichoblatta semisulcata —Princis, 1964: 209.
Pseudoglomeris angustifolia (Wang & Che, 2011)—Li et al. 2018: 27.
Pseudoglomeris (Glomerexis) semisulcata Hanitsch, 1924—Li 2021: 9.

##### Material examined.

China • 4 males & 5 females; Yunnan Province, Dali Bai Autonomous Prefecture, Yangcen 2332 m elev.; 26.4637°N, 99.8016°E; 23 Mar. 2024; Yi-Feng Liu, Peng-Hui Hu, Sen Chen, Tu-Nan Zhou leg; SWU-B-BB104-1 to 9 • 1 male & 2 females; Yunnan Province, Diqing Tibetan Autonomous Prefecture, Haba Village, Mianshaba; 15 Jul. 2024; Lu Qiu leg; SWU-B-BB104-10 to 12 • 1 male; Yunnan Province, Dali Bai Autonomous Prefecture, Qiaohou Town; 18 Jul. 2021; Lu Qiu leg; SWU-B-BB104-13.

**Figure 6.**
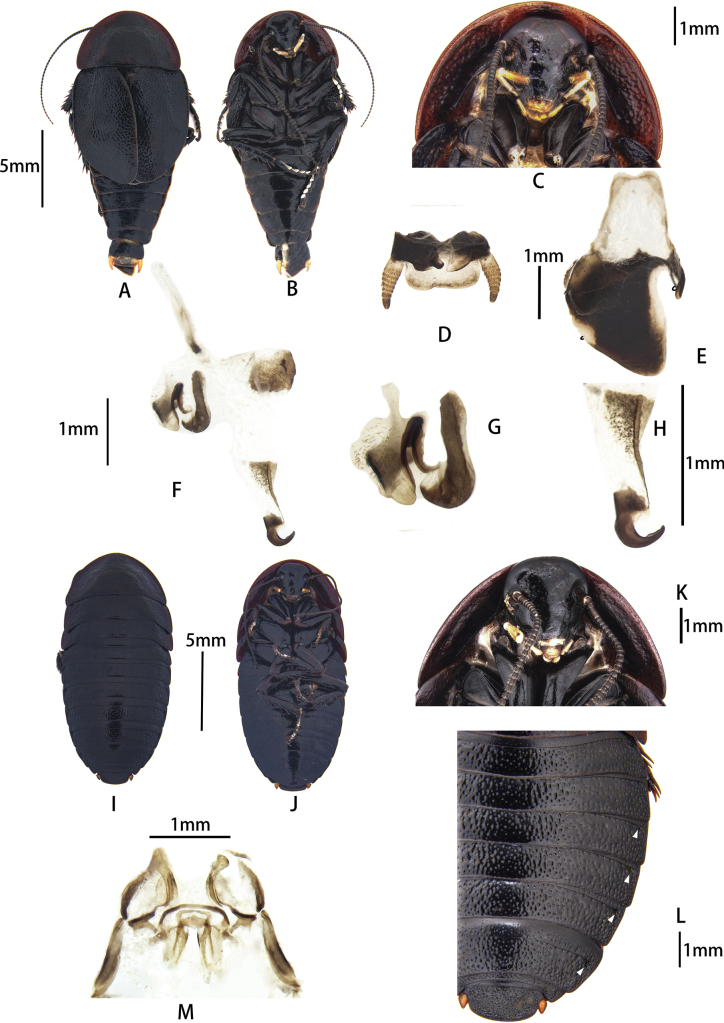
Pseudoglomeris (Glomerexis) semisulcata Hanitsch, 1924 from Dali Bai Autonomous Prefecture, Yangcen. A–H. Male: A. Male, dorsal view; B. Male, ventral view; C. Male pronotum, ventral view; D. Supra-anal plate, ventral view; E. Subgenital plate, dorsal view, missing parts are filled in with black lines; F. Male genitalia, dorsal view; G. Cleft phallomere, dorsal view; H. Hook-like phallomere, dorsal view; I–M. Female: I. Female, dorsal view; J. Female, ventral view; K. Female pronotum, ventral view; L. Female, arrowheads indicate pits; M. Main components of female external genitalia.

##### Supplementary Description.

**Figure 7.**
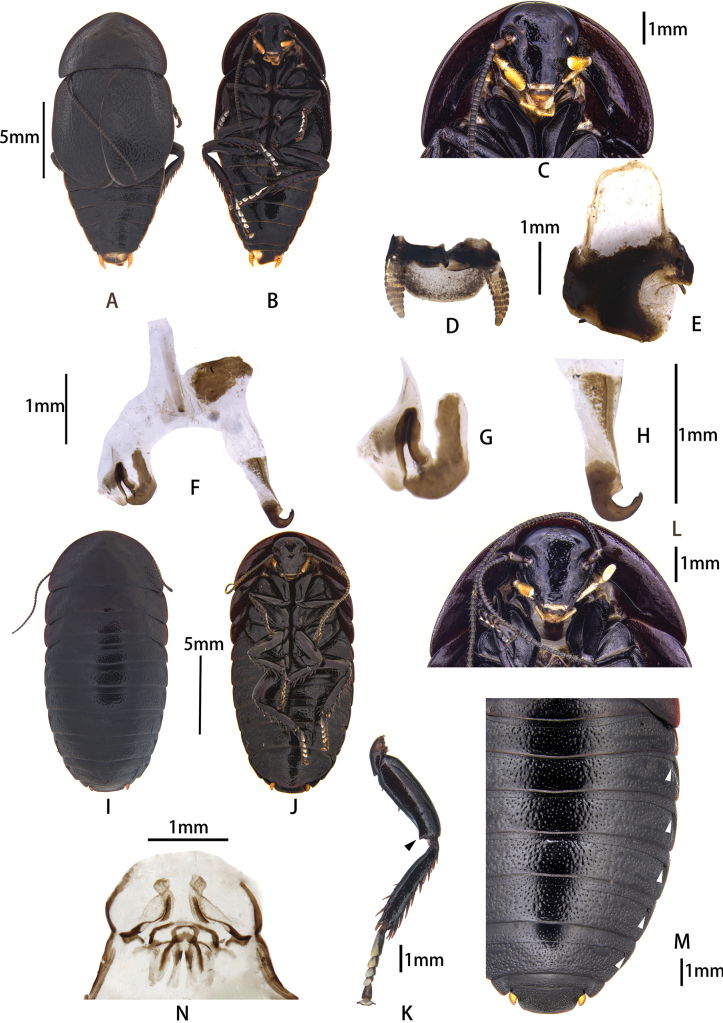
Pseudoglomeris (Glomerexis) angustifolia (Wang & Che, 2011) from Lijiang, Dongshan Lisu Yi Township. A–H. Male: A. Male, dorsal view; B. Male, ventral view; C. Male pronotum, ventral view; D. Supra-anal plate, ventral view; E. Subgenital plate, dorsal view; F. Male genitalia, dorsal view; G. Cleft phallomere, dorsal view; H. Hook-like phallomere, dorsal view; I–N. Female: I. Female, dorsal view; J. Female, ventral view; K. Hind leg, arrowhead indicates sharp projection; L. Female, arrowheads indicate pits; M. Main components of female external genitalia.

**Male.** The tibiae of both the middle and hind legs have a sharp projection at the apex of the ventral surface (Fig. 7K). The hind and middle legs each possess one spine on the posterior margin of the femur and the base.

##### Diagnosis.

This species is similar to P. (G.) angustifolia, and the females of these two species are difficult to distinguish. However, the males can be differentiated by the deep narrow incision near right stylus on the subgenital plate of P. (G.) semisulcata.

#### ﻿ Pseudoglomeris (Glomerexis) angustifolia

Taxon classificationAnimaliaBlattodeaBlaberidae

(Wang & Che, 2011)

E87DBD07-5DED-512F-9CE7-50002648CF80

Fig. 7

Glomerexis angustifolia Wang & Che, 2011: 368.
Pseudoglomeris angustifolia (Wang & Che, 2011)—Li et al. 2018: 281. Pseudoglomeris (Glomerexis) semisulcata Hanitsch, 1924—Li 2021: 9.

##### Material examined.

China • 3 males & 6 females; Yunnan Province, Lijiang, Dongshan Lisu Yi Township, 2778 m elev.; 26.3454°N, 100.8987°E; 27 Apr. 2024; Yi-Feng Liu, Peng-Hui Hu, Sen Chen, Tu-Nan Zhou leg; SWU-B-BB76-1 to 9 • 2 males & 10 females; Yunnan Province, Lijiang, Da’an Yi Naxi Township, 3024 m elev.; 26.7323°N, 100.4729°E; 25 Apr. 2024; Yi-Feng Liu, Peng-Hui Hu, Sen Chen, Tu-Nan Zhou leg; SWU-B-BB76-10 to 21.

##### Supplementary description.

**Male.** The tibiae of both the middle and hind legs feature a sharp projection at the apex of the ventral surface (Fig. 7K). The hind and middle legs each possess one spine on the posterior margin of the femur and the base.

##### Diagnosis.

Refer to the diagnosis of P. (G.) semisulcata.

## ﻿Discussion

This study successfully resolves sexual pairings for six species previously documented from single-sex specimens, including validation of three out of the four “unconfirmed sexual associations” originally noted by Li et al. (2018). Together with the four species that are paired (Li et al. 2018), 10 of the 14 species from China are recognized from both sexes. All species were successfully paired by observing both females and males from the same shelter (under the bark), while nymphs were reared in an artificial environment to facilitate pairing. *Pseudoglomeris* species can be distinguished by their external features, except for the females of P. (G.) semisulcata and P. (G.) angustifolia, as well as the males of P. (P.) montshadskii and P. (P.) dubia. We examined the main components of the female external genitalia, which were found to be simple and variable. Only P. (P.) sculpta exhibits a unique characteristic: the first valvifer arm (gonocoxa; Fig. 2O) has a small fold on the right side, often separating the sclerite; this feature is also present in P. (P.) aerea (Li et al. 2018). Consequently, distinguishing species based solely on female external genitalia is challenging. Similarly, male genitalia are simplified and variable, complicating species differentiation. Therefore, it is essential to integrate geographic distribution data, general morphological traits, and the external genital features of both female and male for an accurate identification of *Pseudoglomeris* species.

## Supplementary Material

XML Treatment for Pseudoglomeris

XML Treatment for Pseudoglomeris (Pseudoglomeris) sculpta

XML Treatment for Pseudoglomeris (Pseudoglomeris) validamoderata

XML Treatment for Pseudoglomeris (Pseudoglomeris) fallax

XML Treatment for Pseudoglomeris (Pseudoglomeris) beybienkoi

XML Treatment for Pseudoglomeris (Glomerexis) semisulcata

XML Treatment for Pseudoglomeris (Glomerexis) angustifolia

## ﻿Additional information

### Use of AI

No use of AI was reported.

### Author ORCIDs

Yi-Feng Liu https://orcid.org/0000-0001-5792-8481

Tu-Nan Zhou https://orcid.org/0009-0003-7902-4071

Sen Chen https://orcid.org/0009-0008-7694-7504

Zong-Qing Wang https://orcid.org/0000-0001-9413-1105

### Data availability

All of the data that support the findings of this study are available in the main text.
